# Maternal dietary manganese protects chick embryos against maternal heat stress via epigenetic-activated antioxidant and anti-apoptotic abilities

**DOI:** 10.18632/oncotarget.20804

**Published:** 2017-09-11

**Authors:** Yongwen Zhu, Lin Lu, Xiudong Liao, Wenxiang Li, Liyang Zhang, Cheng Ji, Xi Lin, Hsiao-Ching Liu, Jack Odle, Xugang Luo

**Affiliations:** ^1^ Mineral Nutrition Research Division, Institute of Animal Science, Chinese Academy of Agricultural Sciences, Beijing 100193, China; ^2^ College of Animal Science, South China Agricultural University, Guangzhou 510000, China; ^3^ College of Animal Science and Technology, China Agricultural University, Beijing 100193, China; ^4^ Department of Animal Science, North Carolina State University, Raleigh, NC 27695, USA

**Keywords:** epigenetics, maternal environmental hyperthermia, manganese superoxide dismutase, apoptosis, chick embryo

## Abstract

Maternal heat stress induced the aberrant epigenetic patterns resulting in the abnormal development of offspring embryos. It is unclear whether maternal dietary manganese supplementation as an epigenetic modifier could protect the chick embryonic development against maternal heat stress via epigenetic mechanisms. To test this hypothesis using an avian model, a completely randomized design with a 2 (maternal normal and high environmental temperatures of 21 and 32°C, respectively) × 3 (maternal dietary manganese sources, the control diet without manganese supplementation and the control diet + 120 mg/kg as either inorganic or organic manganese) factorial arrangement was adopted. Maternal environmental hyperthermia increased mRNA expressions of heat shock proteins 90 and 70, cyclin-dependent kinase 6 and B-cell CLL/lymphoma 2-associated X protein displaying oxidative damage and apoptosis in the embryonic heart. Maternal environmental hyperthermia impaired the embryonic development associated with the alteration of epigenetic status, as evidenced by global DNA hypomethylation and histone 3 lysine 9 hypoacetylation in the embryonic heart. Maternal dietary manganese supplementation increased the heart anti-apoptotic gene B-cell CLL/lymphoma 2 expressions under maternal environmental hyperthermia and manganese superoxide dismutase enzyme activity in the embryonic heart. Maternal dietary organic Mn supplementation effectively eliminated the impairment of maternal environmental hyperthermia on the embryonic development. Maternal dietary manganese supplementation up-regulated manganese superoxide dismutase mRNA expression by reducing DNA methylation and increasing histone 3 lysine 9 acetylation of its promoter. It is suggested that maternal dietary manganese addition could protect the chick embryonic development against maternal heat stress via enhancing epigenetic-activated antioxidant and anti-apoptotic abilities.

## INTRODUCTION

Maternal heat stress can impair productive performance and induce abnormal development in embryos and death in animals [1, 2]. Excessive reactive oxygen species (ROS) from maternal heat stress can enhance embryonic oxidative stress in bovine [3] and porcine [4] species. Exposure of embryos to oxidative stress can induce DNA damage [4, 5] and increase apoptosis [6], leading to developmental arrest or embryonic death in mammals [4, 7]. Oxidative damage from maternal stresses led to global DNA hypomethylation [8, 9] and regional DNA hypermethylation interacting with histone deactylases [10]. The alteration of the epigenetic status was associated with abnormal development and embryo death *in vitro* [11]. Epigenetic changes can further block or silence expression of genes associated with growth and differentiation [12] and genes associated with antioxidant capability [13]. Therefore, it is speculated that aberrant patterns of DNA methylation and histone acetylation induced by maternal heat stress could cause abnormal embryo development by altering imprinted gene expression using a poultry model.

Antioxidants, such as glutathione [14] and vitamins E and C [15], as free radical scavengers, reduce deleterious effects of the increased ROS on the embryonic development under heat shock. The addition of superoxide dismutase to the embryo culture medium can release embryos from the developmental arrest induced by oxidative stress [16, 17]. Manganese (Mn) functioning as a metal cofactor of the metalloenzyme Mn superoxide dismutase (MnSOD) enhanced the antioxidant ability of scavenging excessive ROS [18]. *In vivo*, Mn addition to the Mn-deficient basal diets increased MnSOD activity and mRNA expression in the heart [19–23], and reduced lipid peroxidation in broilers [24]. Moderately chelated organic Mn was the most effective in augmenting these effects [19, 22]. The MnSOD expressions were down-regulated by hypermethylation of CpG islands at *MnSOD* promoter in immortalized fibroblasts [25], multiple myeloma cells [26] and human breast cancer cells [13]. The alteration of epigenetic status of maternal mineral nutrition in deficiency or excess have been extensively studied in embryos [27–30]. Dietary selenium [29] and zinc [30] with a particular emphasis on their antioxidant properties were considered to protect against oxidative stress via epigenetic modifications. Therefore, the objective of the present study was to investigate whether the addition of maternal dietary Mn as an epigenetic modifier could potentially protect chick offspring embryos against maternal heat stress-induced oxidative damage via enhancing antioxidant and anti-apoptotic abilities and epigenetic modifications.

## RESULTS

**Figure 1:**
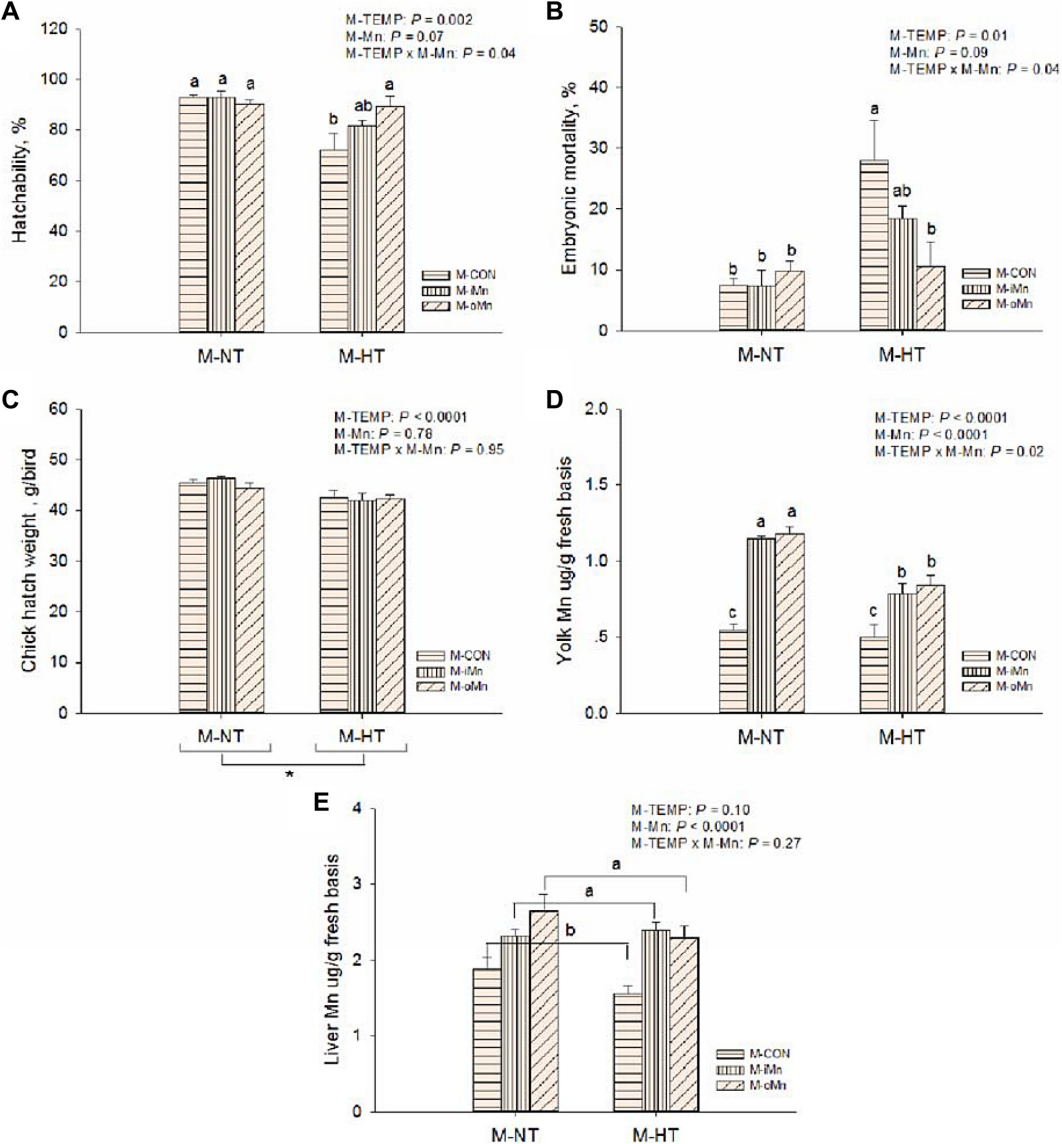
Effects of maternal environmental temperature and dietary Mn on the embryonic development and Mn contents in yolk and liver Hatchability (**A**) and embryonic mortality (**B**) was expressed as percentages in the total fertile eggs per replicate each maternal treatment. Chick hatch weight (**C**) was measured as per replicate each maternal treatment. Yolk (**D**) and liver (**E**) Mn contents were measured based on fresh basis. The data of Mn contents in yolk and liver from the M-CON and M-iMn groups under M-NT as the control groups in the present study have been published in our previous study [31]. Based on the 2-way ANOVA analyses, “_*_” means significant differences at *P <* 0.05 between M-NT (*n* = 18) and M-HT (*n* = 18) as determined by a main effect of maternal environmental temperature; lacking common letters (a, b or c) means significant differences at *P* *<* 0.05 between grouped bars (maternal dietary Mn sources, *n* = 12) or between single bars (individual treatments, *n* = 6) as determined by a main effect of maternal dietary Mn or their interaction. All values are expressed as means ± SE.

**Figure 2:**
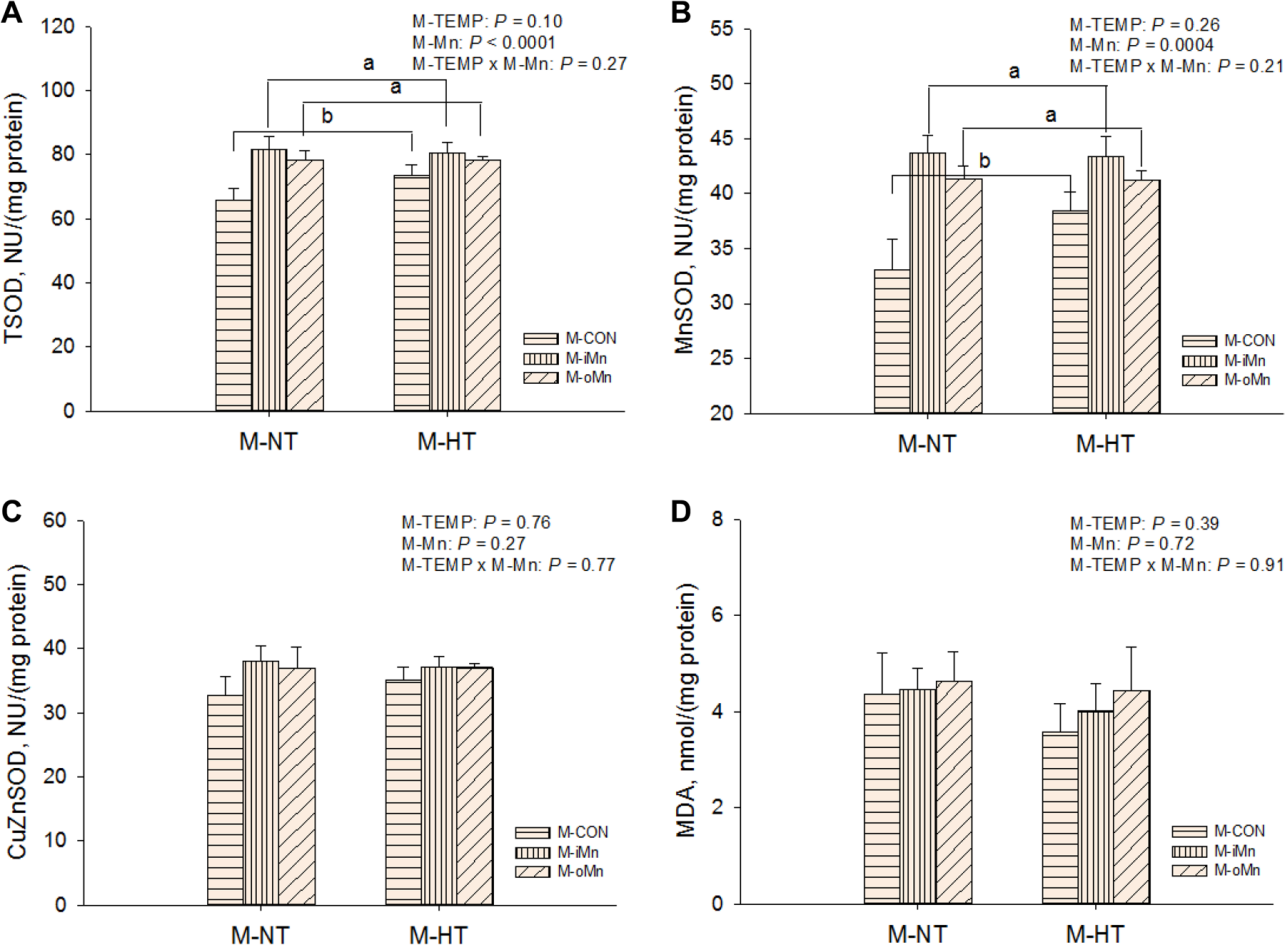
Effects of maternal environmental temperature and dietary Mn on antioxidant ability in the embryonic heart The TSOD activity (**A**), MnSOD (**B**), CuZnSOD activity (**C**) and MDA content (**D**) were used to assess antioxidant ability in the embryonic heart. The data of the above indices from the M-CON and M-iMn groups under M-NT as the control groups in the present study have been published in our previous study [31]. Based on the 2-way ANOVA analyses, lacking common letters (a or b) means significant differences at *P* < 0.05 between grouped bars (maternal dietary Mn sources, *n* = 12) as determined by a main effect of maternal dietary Mn. All values are expressed as means ± SE.

**Figure 3:**
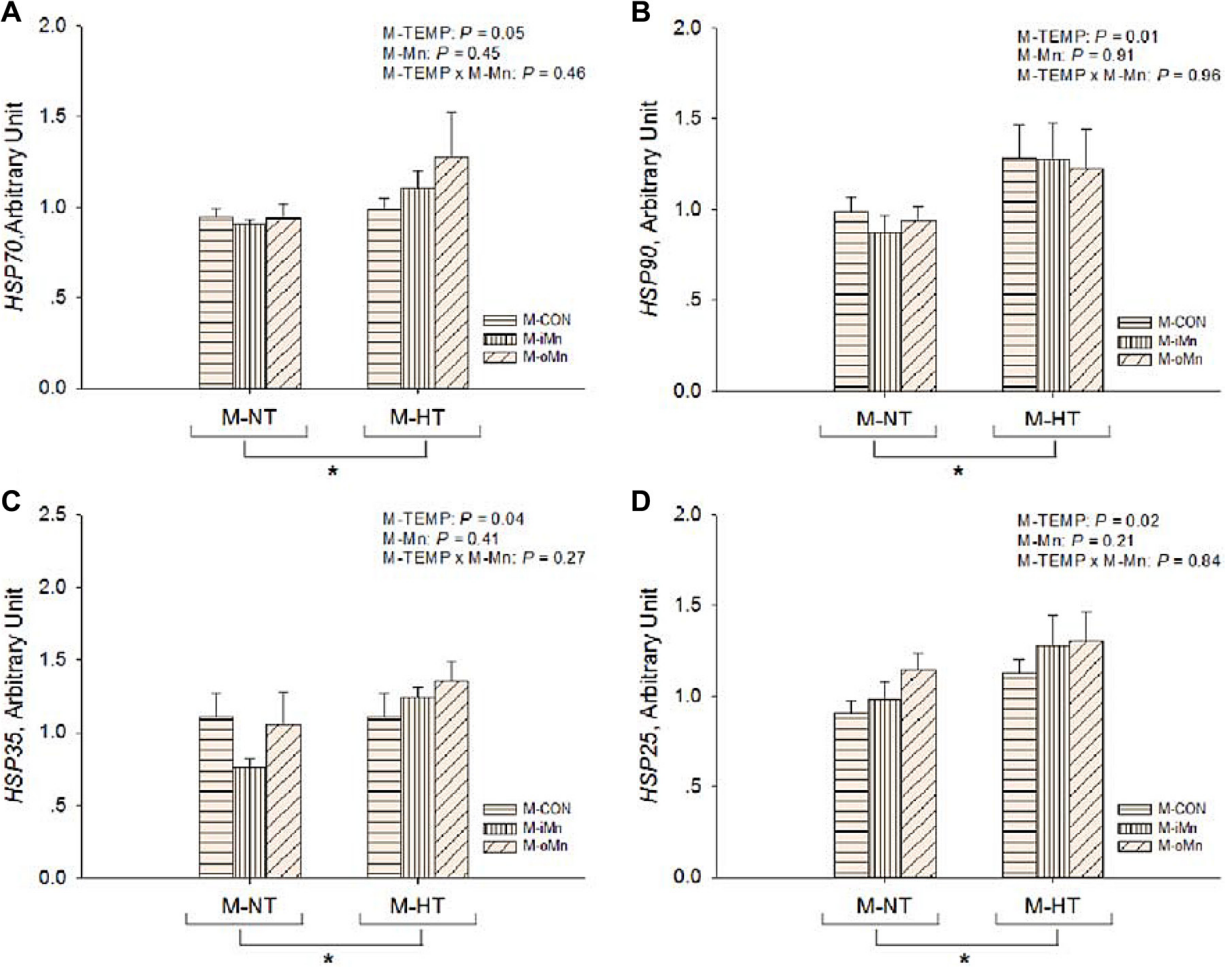
Effects of maternal environmental temperature and dietary Mn on heat shock protein gene mRNA expressions in the embryonic heart The mRNA expressions of *HSP90* (**A**), *HSP70* (**B**), *HSP35* (**C**) and *HSP25* (**D**) genes related with heat stress were determined in the embryonic heart. The geometric mean of internal references, *β-actin* and *GAPDH*, was used to normalize the expression of target genes. The data of *HSP70* and *HSP90* mRNA expressions from the M-CON and M-iMn groups under M-NT as the control groups in the present study have been published in our previous study [31]. Based on the 2-way ANOVA analyses, “_*_” means significant differences at *P* ≤ 0.05 between M-NT (*n* = 18) and M-HT (*n* = 18) as determined by a main effect of maternal environmental temperature. All values are expressed as means ± SE.

**Figure 4:**
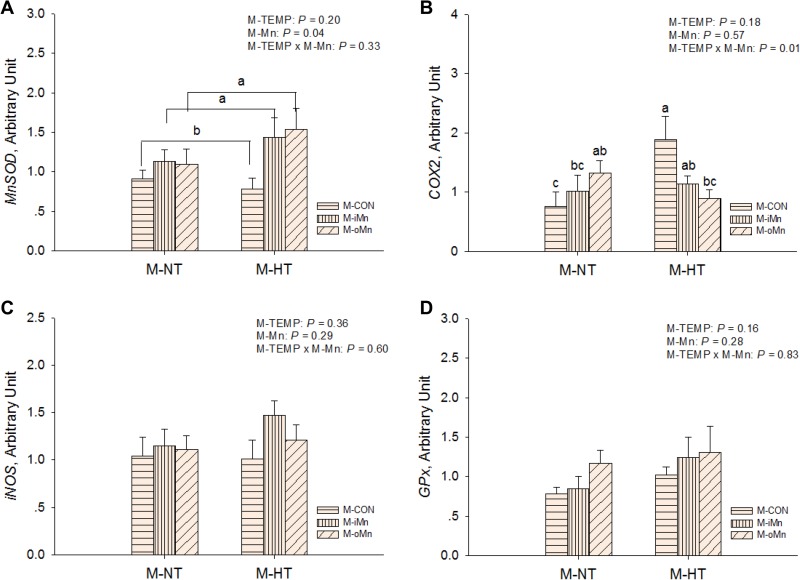
Effects of maternal environmental temperature and dietary Mn on redox-related gene mRNA expressions in the embryonic heart The mRNA expressions of *MnSOD* (**A**), *COX2* (**B**), *iNOS* (**C**) and *GPx* (**D**) genes related with oxidative damage were determined in the embryonic heart. The geometric mean of internal references, *β-actin* and *GAPDH*, was used to normalize the expression of target genes. The data of *MnSOD* mRNA expressions from the M-CON and M-iMn groups under M-NT as the control groups in the present study have been published in our previous study [31]. Based on the 2-way ANOVA analyses, lacking common letters (a, b or c) means significant differences at *P* *<* 0.05 between grouped bars (maternal dietary Mn sources, *n* = 12) or between single bars (individual treatments, *n* = 6) as determined by a main effect of maternal dietary Mn or their interaction. All values are expressed as means ± SE.

To address the roles of the maternal dietary inorganic and organic Mn sources in the development of offspring embryos from the heat stressed broiler breeders as well as the deep mechanisms involved, the related data (see the captions in Figures 1–4 and Supplementary Figure 1) collected from chick embryos delivered from the control broiler breeders in our previous published study [31] were used for statistical analyses in the present study. The previous study [31] was conducted at the same time and facility as this study, and the broiler breeders raised at the normal temperature with a Mn-unsupplemented diet or the basal diet supplemented with the inorganic Mn were treated as the control maternal groups for the present study in accordance with the requirements of the Animal Welfare Committee of the Institute of Animal Science, Chinese Academy of Agricultural Sciences.

### Hatchability, embryonic mortality and chick hatch weight

Hatchability (Figrue 1A), embryonic mortality (Figrue 1B), and chick hatch weight (Figrue 1C) were affected (*P* ≤ 0.01) by maternal environmental temperatures (M-TEMP). Compared to NT, maternal high temperature (M-HT) decreased (*P* < 0.003) hatchability, hatch weight of chicks, and increased (*P* < 0.02) embryonic mortality. There were significant interactions (*P* < 0.05) in hatchability and embryonic mortality between M-TEMP and maternal dietary Mn (M-Mn). Under maternal normal temperature (M-NT), M-Mn did not affect (*P* > 0.28) on hatchability and embryonic mortality, while under HT, compared to the maternal control basal diet (M-CON), maternal dietary supplementation with the organic Mn (M-oMn) significantly increased (*P* < 0.05) hatchability and significantly reduced (*P* < 0.05) mortality of offspring chick embryos to the similar levels of those in the M-NT groups, and no differences (*P* > 0.10) were observed between the maternal basal diet supplemented with the inorganic Mn (M-iMn) and M-CON or between the two Mn sources.

### Manganese contents in egg yolk and the embryonic liver

To determine whether maternal environmental temperature and dietary Mn could affect Mn deposition in offspring embryonic tissues, Mn content was measured in egg yolk (Figrue 1D) and the embryonic liver (Figrue 1E) from each maternal treatment. The Mn content in egg yolk was influenced (*P* < 0.02) by M-TEMP, M-Mn and their interaction. Compared to M-NT, as for M-CON, M-HT did not reduce (*P* > 0.61) Mn content in egg yolk, however, as for either M-iMn or M-oMn, M-HT reduced (*P* < 0.0002) Mn content in egg yolk. The Mn contents in liver were influenced (*P* < 0.0001) by M-Mn, but not (*P* ³Unknown 0.10) by M-TEMP or their interaction. M-Mn increased (*P* < 0.0002) Mn content in egg yolk 2-fold compared to the control diet, and subsequently increased (*P* < 0.0001) Mn content in the embryonic liver, and no differences (*P* > 0.21) were observed between the two Mn sources.

### Antioxidant indices in the embryonic liver and heart

To determine whether maternal dietary Mn addition, particularly the organic Mn, could attenuate oxidative damage in offspring embryos from maternal HT, the activities of the total superoxide dismutase (TSOD), MnSOD and copper zinc superoxide dismutase (CuZnSOD, Figrue 2C) as well as malonaldehyde (MDA, Figrue 2D) contents in the heart and liver of embryos were measured. There were no interactions (*P* > 0.20) between M-TEMP and M-Mn in any of these indices. The liver TSOD (Supplementary Figure 1A), MnSOD (Supplementary Figure 1B) and CuZnSOD (Supplementary Figure 1C) activities and MDA contents in liver (Supplementary Figure 1D) and heart (Figrue 2D) were not influenced (*P* > 0.26) by M-TEMP and M-Mn. The activities of TSOD (Figrue 2A) and MnSOD (Figrue 2B) in the heart of embryos were affected (*P* < 0.003) by M-Mn, but not (*P* > 0.25) by M-TEMP. The activities of TSOD and MnSOD in the heart of embryos from M-iMn and M-oMn were higher (*P* < 0.0004) than those from M-CON, and no difference (*P* > 0.82) was observed between the inorganic and organic Mn sources.

### HSPs, antioxidant and apoptosis related genes mRNA expression in the embryonic heart

The mRNA abundances of HSP genes as sensitive biomarkers of oxidative damage, such as *heat shock proteins 70, 90, 35 and 25* (HSP70, 90, 35, and 25, Figure 3A–3D) in the embryonic heart, were detected by RT-qPCR. Compared to M-NT, M-HT increased (*P* ≤ 0.05) the mRNA expressions of all measured HSPs in heart. No effects (*P* > 0.20) of M-Mn and interactions between M-TEMP and M-Mn were detected. We further measured mRNA expressions of other redox-related genes connected with mitochondrial function, such as *MnSOD, cyclooxygenase 2 (*COX2*), inducible nitric oxide synthase (*iNOS*),* and *glutathione peroxidase* (GPx) in the embryonic heart (Figure 4A–4D). The mRNA expressions of *iNOS* and *GPx* were not affected (*P* > 0.15) by M-TEMP, M-Mn or their interaction. However, heart *MnSOD* mRNA expression was affected (*P* < 0.05) by M-Mn, but not (*P* > 0.19) by M-TEMP and their interaction. The *MnSOD* mRNA expression in the heart of embryos from hens fed diets supplemented with either inorganic Mn or organic Mn was higher (*P* < 0.05) than that from M-CON with no difference (*P* > 0.85) between the two Mn sources. A significant interaction (*P* < 0.02) between M-TEMP and M-Mn was detected in the heart *COX2* mRNA. Compared to M-NT, M-HT increased (*P* < 0.003) *COX2* mRNA expression in the heart of embryos from M-CON, but had no effect (*P* > 0.24) on that from either M-iMn or M-oMn. Under M-HT, both M-iMn and M-oMn decreased (*P* < 0.05) heart *COX2* mRNA expression compared to M-CON with no difference (*P* > 0.27) between M-iMn and M-oMn.

**Figure 5:**
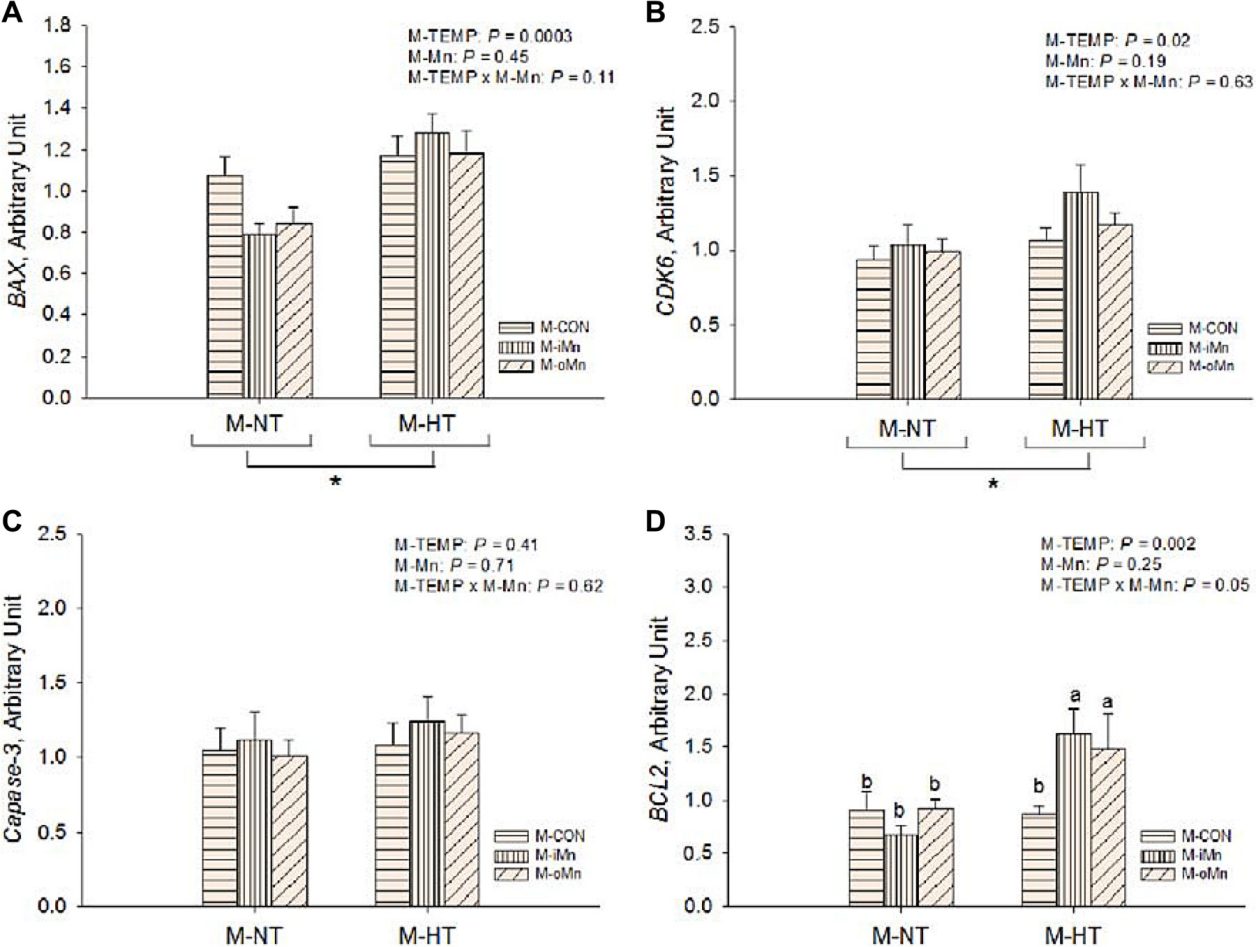
Effects of maternal environmental temperature and dietary Mn on anti-apoptosis related gene mRNA expressions in the embryonic heart The mRNA expressions of *BAX* (**A**), *CDK6* (**B**), *caspase-3* (**C**) and *BCL2* (**D**) genes related with apoptosis damage were determined in the embryonic heart. The geometric mean of internal references, *β-actin* and *GAPDH*, was used to normalize the expression of target genes. Based on the 2-way ANOVA analyses, “_*_” means significant differences at *P* < 0.05 between M-NT (*n* = 18) and M-HT (*n* = 18) as determined by a main effect of maternal environmental temperature; lacking common letters (a or b) means significant differences at *P* = 0.05 between single bars (individual treatments, *n* = 6) as determined by their interaction. All values are expressed as means ± SE.

To determine whether maternal dietary supplementation with Mn could reduce the deleterious effect of maternal heat stress on apoptosis or cell proliferation of embryos, the mRNA expressions of *B-cell CLL/lymphoma 2 (*BCL2*), BCL2-associated X protein (*BAX*), caspase-3 (*Casp3) and *cyclin-dependent kinase 6 (*CDK6) in the embryonic heart were measured (Figure 5A–5D). The mRNA expressions of *BAX* and *CDK6* were affected (*P* < 0.03) by M-TEMP, but not (*P* > 0.10) by M-Mn and their interaction. Compared to M-NT, M-HT increased (*P* < 0.03) mRNA expressions of *BAX* and *CDK6*. M-Mn, M-TEMP and their interaction did not affect (*P* > 0.40) on the heart *Casp3* mRNA expression. Heart *BCL2* mRNA expression was affected (*P* ≤ 0.05) by M-TEMP and interaction between M-Mn and M-TEMP, but not (*P* > 0.24) by M-Mn. Under M-NT, heart *BCL2* mRNA expression was not affected (*P* > 0.28) by M-Mn, while under M-HT, heart *BCL2* mRNA expression from either M-iMn or M-oMn was higher (*P* < 0.002) than that from M-CON, and no difference (*P* > 0.55) was observed between the M-iMn and M-oMn.

### Protein expression in the embryonic heart

**Figure 6:**
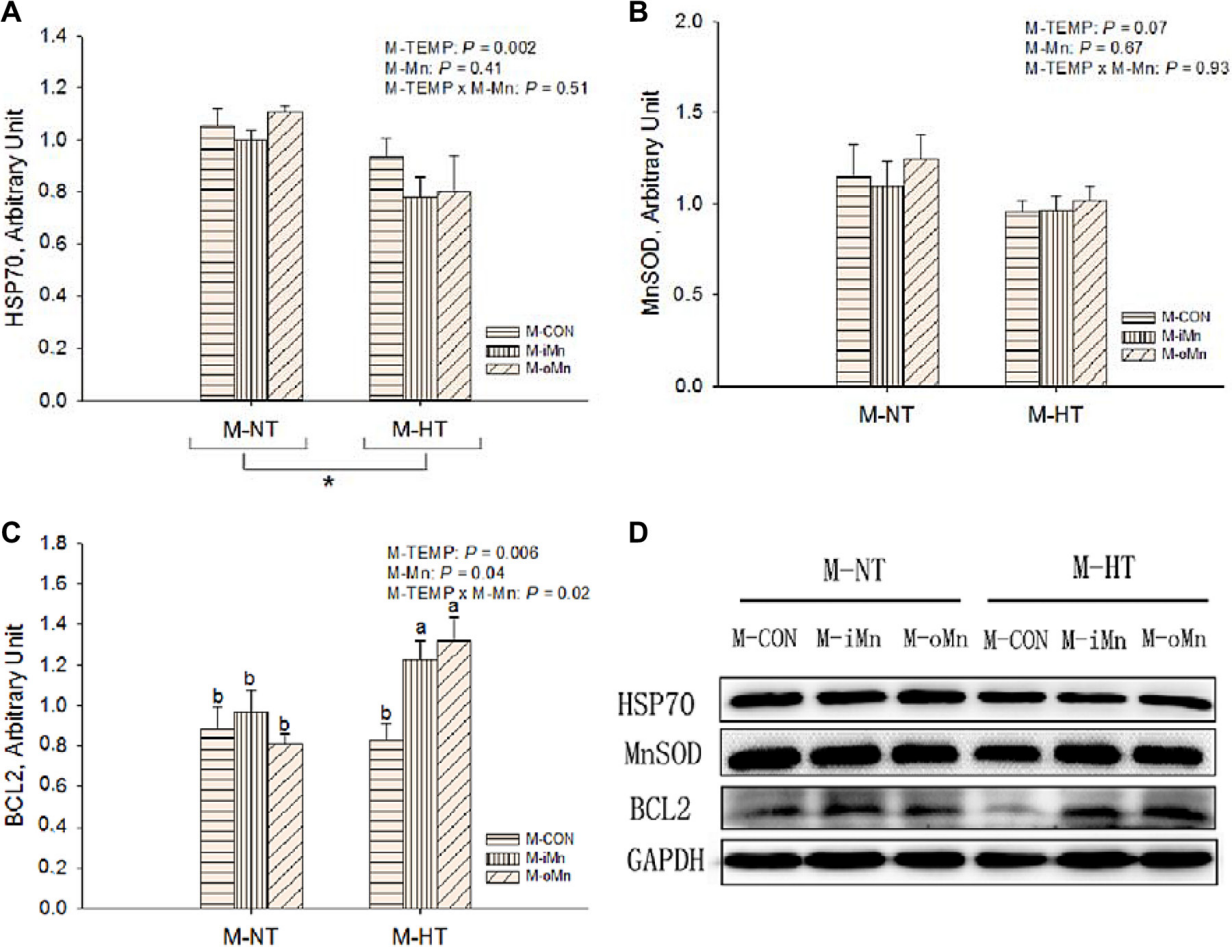
Effects of maternal environmental temperature and dietary Mn on HSP70 (A), MnSOD (B) and BCL2 (C) protein expressions in the embryonic heart The GAPDH was selected to normalize the expression of target protein. (**D**) Representative immunoblots of the indicated proteins were shown. The data of HSP70 protein expressions from the M-CON and M-iMn groups under M-NT as the control groups in the present study have been published in our previous study [31]. Based on the 2-way ANOVA analyses, “_*_” means significant differences at *P* < 0.05 between M-NT (*n* = 18) and M-HT (*n* = 18) as determined by a main effect of maternal environmental temperature; lacking common letters (a or b) means significant differences at *P* < 0.05 between single bars (individual treatments, *n* = 6) as determined by their interaction. All values are expressed as means ± SE.

**Figure 7:**
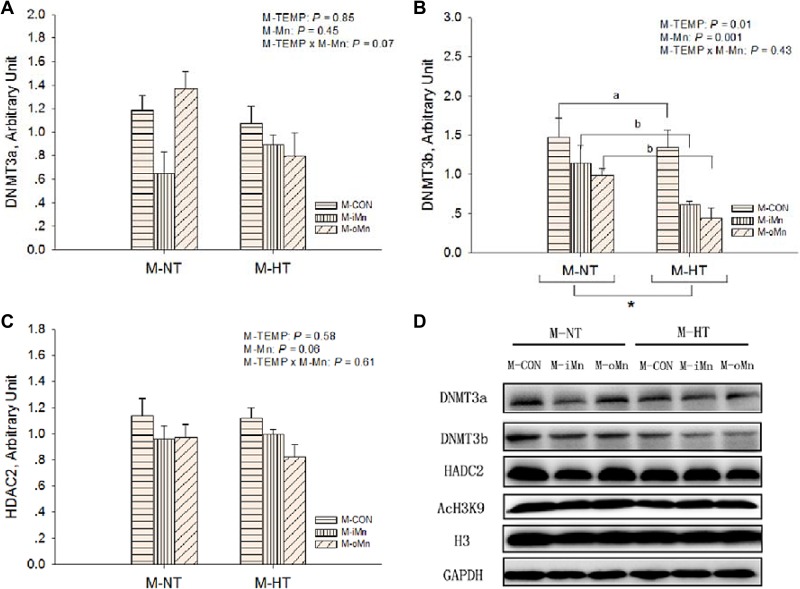
Effects of maternal environmental temperature and dietary Mn on epigenetic related protein expressions in the embryonic heart The GAPDH was selected to normalize the expression of target proteins of DNMT3a (**A**), DNMT3b (**B**) and HDAC2 (**C**). (**D**) Representative immunoblots of the indicated proteins were shown. Based on the 2-way ANOVA analyses, “_*_” means significant differences at *P* < 0.05 between M-NT (*n* = 18) and M-HT (*n* = 18) as determined by a main effect of maternal environmental temperature; lacking common letters (a or b) means significant differences at *P* < 0.05 between grouped bars (maternal dietary Mn sources, *n* = 12) as determined by a main effect of maternal dietary Mn. All values are expressed as means ± SE.

We further detected protein expressions of HSP70 (Figure 6A), MnSOD (Figure 6B), BCL2 (Figure 6C), DNA methyltransferases 3a and 3b (DNMT3a and DNMT3b, Figure 7A–7B), and histone deacetylase 2 (HDAC2, Figure 7C) in the embryonic heart. Those representative immunoblots were listed in Figure 6D and 7D, respectively. Heart HSP70 protein expression was affected (*P* < 0.003) by M-TEMP, but not (*P* > 0.40) by M-Mn and the interaction between M-Mn and M-TEMP. M-HT decreased (*P* < 0.05) heart HSP70 protein expression compared to M-NT. Heart MnSOD protein expression was not affected by M-TEMP (*P* = 0.07), M-Mn (*P* = 0.67) and their interaction (*P* = 0.93). Heart BCL2 protein expression was affected (*P* < 0.05) by M-TEMP, M-Mn and their interaction. Under M-NT, heart BCL2 protein expression was not affected (*P* > 0.53) by M-Mn, while under M-HT, heart BCL2 protein expression from either M-iMn or M-oMn was higher (*P* < 0.008) than that from M-CON, and no difference (*P* > 0.47) was observed between the two Mn sources. Therefore, the alteration of heart BCL2 protein expression induced by M-TEMP and M-Mn was well correlated with that of its mRNA expression. Heart protein expressions of DNMT3a and HDAC2 were not affected (*P* > 0.05) by M-TEMP, M-Mn and their interaction. Heart DNMT3b protein expression was affected (*P* < 0.02) by M-TEMP and M-Mn, but not (*P* > 0.42) by the interaction between M-TEMP and M-Mn. Compared to M-NT, M-HT decreased (*P* = 0.01) heart DNMT3b protein expression. Compared to M-CON, either M-iMn or M-oMn decreased (*P* < 0.006) heart DNMT3b protein expression with no difference (*P* > 0.33) between the two Mn sources.

### Global levels of DNA methylation and histone 3 lysine 9 (H3K9) acetylation

**Figure 8:**
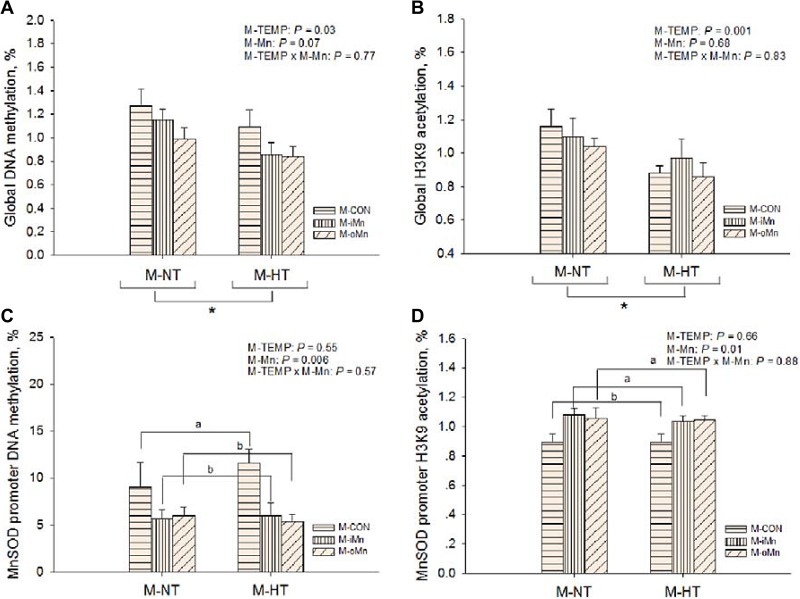
Effects of maternal environmental temperature and dietary Mn on DNA methylation and H3K9 acetylation in the embryonic heart The global levels of DNA methylation (**A**) and H3K9 acetylation (**B**) were determined using ELISA and western-blot methods, respectively. The *MnSOD* promoter DNA methylation (**C**) and H3K9 acetylation (**D**) were determined using methylated DNA immunoprecipitation and chromatin immunoprecipitation methods, respectively. Based on the 2-way ANOVA analyses, “_*_” means significant differences at *P* < 0.05 between M-NT (*n* = 18) and M-HT (*n* = 18) as determined by a main effect of maternal environmental temperature; lacking common letters (a or b) means significant differences at *P* < 0.05 between grouped bars (maternal dietary Mn sources, *n* = 12) as determined by a main effect of maternal dietary Mn. All values are expressed as means ± SE.

To determine whether epigenetic status in offspring embryos was altered by M-TEMP and M-Mn, the global level of DNA methylation (Figure 8A) and the relative degree of histone H3K9 acetylation (Figure 8B) after normalization to total histone H3 level in the embryonic heart were measured. Global levels of both DNA methylation and histone H3K9 acetylation were affected (*P* < 0.04) by M-TEMP, but not by M-Mn (*P* > 0.06) and the interaction (*P* > 0.76) between M-TEMP and M-Mn. M-HT had lower (*P* < 0.05) global levels of both DNA methylation and histone H3K9 acetylation than M-NT.

### The DNA methylations and H3K9 acetylations in MnSOD promoter

According to the alteration of the epigenetic status, we further examined the DNA methylations (Figure 8C) and histone H3K9 acetylations *MnSOD* promoter (Figure 8D) in the embryonic heart by a combination of immunoprecipitation and real-time PCR, which was related to a regulation of their transcriptional activity. M-Mn affected (*P* < 0.02) DNA methylation and histone H3K9 acetylation of *MnSOD* promoter. Compared to M-CON, both M-iMn and M-oMn decreased (*P* < 0.007) DNA methylation of *MnSOD* promoter, but increased (*P* < 0.006) its histone H3K9 acetylation with no difference (*P* > 0.86) between the two Mn sources. No interactions (*P* > 0.29) were detected between M-TEMP and M-Mn in the above mentioned indices.

## DISCUSSION

As reported previously in laying hens [2, 32], maternal heat stress significantly reduced hatchability in the female broiler breeders. Maternal heat stress led to high embryonic mortality and low birth weight, which has been confirmed in mammals [3, 4]. The adverse effect of maternal heat stress on embryonic development might be related to oxidative stress with the increased production of ROS [3, 4]. Excessive ROS can induce damage of lipids, protein and DNA and then lead to developmental arrest or embryonic death [7]. However, in our study, lipid peroxidation was not observed in the chick embryos from maternal heat stress based on the MDA contents in the liver or heart. One reason might be that embryos from the later stage in development have a more complete antioxidant capacity to against oxidative stress. Another reason might be that MDA content in tissues was not a sensitive biomarker for reflecting oxidative damage of the chick embryo during incubation. In fact, ROS not only have a direct effect on cells, but also act as second messengers by regulating key transcription factors that alter gene expressions in the embryo, such as in heat shock factors (HSF) [33] and nuclear factor κB (NF-κB) [34]. Studies have demonstrated the accumulation of ROS increased the binding activity of HSF1 and *HSP70* and *HSP90* mRNA expressions in rat hearts [33, 35]. The increased synthesis of HSPs could be utilized as a sensitive redox biomarker representing oxidative damage [36, 37]. In the present study, maternal heat stress increased mRNA abundance of *HSP90, HSP70, HSP35* and *HSP25* in chick embryonic heart, suggesting that oxidative damage might be induced in chick embryos subjected to maternal heat stress. However, heart HSP70 protein expression was reduced by maternal heat stress, which was opposite to its mRNA expression. In a steady state, the amount of protein expression agrees with the specific mRNA level, depending on the post-transcriptional regulation and protein stability [38]. Therefore, the contrary results between mRNA and protein expressions suggest that maternal heat stress might decrease the translational efficiency of *HSP70* mRNA and the half-life of the HSP70 protein. The HSP70 as a molecular chaperone mediates protein folding, assembly, transport and degradation during embryonic development could block the activation or the activity of the caspases to inhibit apoptosis [39]. The heat-induced oxidative damage with a parallel reduction of HSP70 could enhance apoptosis in chick embryos. Meanwhile, an increased apoptosis-related *BAX* mRNA expression was observed in maternal heat-stressed chick embryos as reported previously [40]. This might be one of the reasons for the high embryonic mortality induced by maternal heat stress.

Glutathione [14] and vitamin E and C [15] as free radical scavengers have been reported to reduce deleterious effects of the increased ROS on developmental embryos *in vitro* under heat shock. The addition of SOD to the embryo culture medium released embryos from the developmental arrest induced by oxidative stress [16, 17]. Manganese functioning as a key component of MnSOD enzyme increased the antioxidant ability in scavenging excessive ROS [18]. Previous studies have proved that supplemental Mn in the Mn-unsupplemented basal diets increased MnSOD activity and mRNA expression in the heart of broilers [19–23]. In the present study, under M-HT, compared to M-CON, dietary supplementation with the organic Mn increased hatchability and decreased embryonic mortality to levels similar to those under M-NT, suggesting that maternal diet with organic Mn supplementation could effectively and completely eliminate the negative effect of maternal heat stress on embryonic development. Our results suggested that the Mn accumulation in the eggs from broiler breeders fed Mn-supplemented diets could be transferred to the developing embryos and subsequently increase liver Mn and activities of heart TSOD and MnSOD in the embryonic regardless of maternal environmental temperature. It was also implied that the embryos from the birds given the Mn-supplemented diets might benefit from the enhanced antioxidant ability and thus be protected from heat stress. The embryonic heart *MnSOD* mRNA expression and activity, but not its protein expression, were elevated by maternal dietary supplementation with either the inorganic or the organic Mn. It was implied that maternal dietary Mn supplementation up-regulated MnSOD expression in the embryonic heart at transcriptional and post-translational levels, but not at a translational level. The results have been confirmed in our previous studies in broilers under thermoneutral conditions [19–23]. The lack of correlation between mRNA and protein abundance implied that other regulatory mechanisms might be involved in the transcription and translation of *MnSOD*, such as the regulations of NF-κB [41] and P53 pathways [42] as well as microRNAs [43]. The bioavailability or efficacy of a nutrient for animals should be evaluated at dietary levels around its requirement level as long as there is a good linear relationship between the responsive index and dietary nutrient level. A series of studies on the bioavailabilties of Mn sources for broilers as estimated at dietary Mn levels around the requirement of broilers have been done, and the results indicate that the moderately chelated organic Mn displayed significantly higher Mn bioavailability or efficacy than inorganic Mn in augmenting heart MnSOD gene expressions and activities in broiler chicks [19–23]. In the present study, the supplemental Mn level of 120 mg/kg in the corn-soybean meal basal diet was selected based on the dietary Mn requirement of broiler breeders [44, 45]. In fact, heat stress could enhance mineral excretion along with lower feed intake and mineral digestibility [46]. Therefore, the supplemental Mn level of 120 mg/kg might be slightly below their requirement level and suitable for evaluating Mn efficacy. However, no differences in efficacy between the two Mn sources were detected in all measured indices of embryos from either M-NT or M-HT. This was probably due to the lack of difference in egg yolk Mn content between the two Mn sources. It is well known that the egg is not a main and sensitive target organ for accumulations of Mn and other minerals in the body, although the majority of Mn in the egg is accumulated in the egg yolk. Therefore, egg yolk Mn content is not a sensitive parameter to detect the differences between the two Mn sources in Mn efficacy for broiler breeders, and the above results would be reasonable and expected since there was no difference between the two Mn sources in egg yolk Mn accumulation closely related with the embryonic Mn metabolism and embryonic development.

Accumulated ROS from oxidative stress in mitochondria can damaged directly the functions of mitochondrial proteins, lipids and DNA, obscuring the operation of the organelles [47]. ROS-triggered mitochondrial dysfunction has also been implicated in the initiations of the transcription of inflammatory gene *COX2* and cell apoptosis [48, 49]. In the present study, maternal heat stress increased *COX2* mRNA expression in the heart of embryos from M-CON, but had no effect on that from either M-iMn or M-oMn. It is implied that maternal dietary supplementation with Mn could provide much better mitochondrial protection against oxidative damage based on the indicator of *COX2* mRNA expression. The MnSOD as the most dominant antioxidant enzyme functions as scavenging free radicals in mitochondria. Animals given Mn-deficient diets developed Mn-responsive abnormalities in heart mitochondrial ultrastructures, accompanied by a reduced MnSOD activity [50]. The addition of MnSOD to the culture medium was more effective in delaying the apoptosis compared with the untreated group [51]. Under maternal heat stress, compared to the M-CON, maternal dietary supplementation with Mn increased anti-apoptotic gene *BCL2* mRNA and protein expressions in the embryonic heart to block or delay apoptosis resulting from oxidative damage. Anti-apoptotic protein BCL2 located primarily at the outer mitochondrial membrane, prevents mitochondrial permeability transition and cytochrome c release [52] via antioxidant pathway [53]. Our results suggested that the induced BCL2 expressions due to maternal dietary Mn addition could increase cellular redox capacity and inhibit cell death by reducing oxidative damage from maternal heat stress. It is implied that maternal dietary supplementation with Mn was potentially in favor of embryonic development related to the protection of mitochondria and anti-apoptosis.

It has been demonstrated that maternal environmental and nutritional stresses influenced the epigenetic status during embryogenesis via DNA methylation and histone modifications [54]. The changes of DNA methylation achieved by de novo methyltransferases (DNMT3a and DNMT3b) could induce the abnormal histone H3 acetylation [10]. Maternal stress, especially oxidative stress, induced aberrant patterns of DNA methylation and histone acetylation in mammals [8]. Both the global DNA hypomethylation and H3K9 hypoacetylation have been detected in the heart of offspring embryos from maternal heat stress. Similar results were observed in cancer cells involving oxidative damage [9, 10]. DNA lesions such as strand breakage and chromosomal rearrangements from generation of ROS have been shown to inhibit catalytic reactions between DNMTs and DNA as well as between HATs and the substrate histone [9]. Additionally, maternal heat stress suppressed DNMT3b protein expression resulting in global DNA hypomethylation in chick embryonic heart. A decrease in DNA methylation was associated with abnormal development and embryo death in embryonic stem cells [54] and rat embryos *in vitro* [11], which was confirmed in chick embryos in the current study. Aberrant epigenetic mechanisms were implicated in modulating gene expression and thus affecting embryonic development [55]. The DNA methylation involves a direct interference with the binding of specific transcriptional enhancers to their promoter and then inhibits gene transcription. In cancer cells, the potential of dietary selenium [27] and zinc [28], with a particular emphasis on their antioxidant properties was thought to protect against in oxidative stress via epigenetic modifications. Therefore, in the present study, we also investigated whether maternal dietary Mn addition could alter epigenetic status and imprinted antioxidant gene expression to protect embryos against maternal heat stress. It has been proven that DNA hypermethylation at the promoter decreased *MnSOD* expressions in breast cancer cells [13], immortalized fibroblasts [25], and multiple myeloma cells [26]. One region of the *MnSOD* promoter CpG methylation level in the heart of chick embryos has been identified to be higher for M-CON than for either M-iMn or M-oMn. The DNA hypermethylation of the *MnSOD* promoter in embryonic heart from M-CON might result in the inhibition of *MnSOD* mRNA expression. This hypermethylation could be associated with the elevated DNMT3b protein expression that we observed. The chick embryos from M-CON with aberrantly increased heart *MnSOD* DNA methylation also displayed a decreased heart histone H3K9 acetylation in the same region of the *MnSOD* gene, which confirmed by the previous study in breast cancer cells [13]. This demonstrated that the chromatin containing methylated DNA is characterized as the absence of acetylation on histone H3. Histone deacetylation was also reported to be associated with gene silencing via alterations in the structure of the nucleosomes or activation of RNA polymerase and other transcription coactivator binding [56]. The above results support that a potential role of maternal dietary Mn addition is to up-regulate the *MnSOD* mRNA expression in the heart of offspring chick embryos via alterations of its DNA methylation and histone H3K9 acetylation potentially.

In conclusion, the results demonstrate that maternal environmental hyperthermia impairs the embryonic development and increases mRNA expressions of *HSPs*, *CDK6* and *BAX*, and induces global DNA hypomethylation and H3K9 hypoacetylation in the embryonic heart. Maternal dietary supplementation with either inorganic Mn or organic Mn enhanced mRNA and protein expression levels of anti-apoptotic gene BCL2 under maternal environmental hyperthermia and MnSOD activity in the embryonic heart. Maternal dietary supplementation of the organic Mn effectively eliminates the adverse effect of maternal heat stress on the embryonic development. Maternal dietary Mn supplementation up-regulates the heart *MnSOD* mRNA expression potentially by reducing DNA methylation and increasing histone H3K9 acetylation of the *MnSOD* promoter regardless of Mn source. It is suggested that maternal dietary Mn addition could protect the chick embryonic development against maternal heat stress via enhancing epigenetic-activated antioxidant and anti-apoptotic abilities.

## MATERIALS AND METHODS

### Experimental design and treatments

This experiment included two consecutive phases of maternal broiler breeders and offspring embryos. Experimental design and treatments for the maternal broiler breeder phase were described in details in our previous study [57]. Briefly, a completely randomized design with a 2 [maternal normal and high environmental temperatures of 21 (M-NT) and 32°C (M-HT), respectively] × 3 [maternal dietary Mn sources, the maternal control basal diet without Mn supplementation (M-CON), the M-CON diet + 120 mg Mn/kg as either the inorganic MnSO_4_·H_2_O (M-iMn) or organic Mn proteinate (M-oMn)] factorial arrangement of treatments was adopted. All eggs collected from the above 6 maternal treatments were incubated at the normal incubation temperature (37.8°C) for the 21-d offspring embryo phase.

### Animals and diets

This study was approved by the Animal Welfare Committee of the Institute of Animal Science, Chinese Academy of Agricultural Sciences. All experimental procedures were carried out in strict accordance with the recommendations in the Guide for the Care and Use of Animals of the Chinese Academy of Agricultural Sciences and approved by the Animal Welfare Committee of the Institute of Animal Science, Chinese Academy of Agricultural Sciences. As reported for the maternal broiler breeder phase in our previous study [57], a total of 144 18-wk-old broiler breeder hens were randomly assigned to 1 of 6 treatments with 6 replicates (4 birds per replicate) for each treatment. All broiler breeders initially were fed a conventional diet for a period of adaption from 18 to 29 wk of age according to Arbor Acres breeder management guides, and then fed a corn-soybean meal basal diet with no Mn addition from 30 to 31 wk of age to further deplete the body's stores of Mn. After Mn depletion, the hens from 3 dietary Mn sources under the normal temperature were kept at 21 ± 1°C, while the birds from 3 dietary Mn sources under the high temperature were reared at 32 ± 1 °C for 9 wks from 32 to 40 wk of age. The composition of the corn-soybean meal basal diet for the Mn-depletion and the experimental stages and the analyzed Mn content in the basal diet (14.3 mg/kg) were the same as described in our previous study [57]. Throughout the experiment, birds were given free access to tap water and the experimental diets. All broiler breeders were inseminated weekly. All eggs from 6 replicates of each of the above 6 maternal treatments were collected during the last 2 wk of the experimental period, and then incubated in the same incubator with 6 egg trays (9TDJ-A, LanTianJiao Electronic Technology company, Beijing, China). All eggs from each replicate of all of the above 6 maternal treatments were placed on one egg tray. The incubator was set at an incubation temperature of 37.8 °C and a relative humidity of 55 to 60%. The clear eggs were removed after candling on d 10 of the incubation (E10). All clear eggs on E10 and non-hatched eggs on E21 were counted, opened, and visually evaluated also to determine the true embryonic mortality. Hatchability and embryonic mortality were expressed as percentages of the total number of fertile eggs of each replicate per maternal treatment.

### Sample collections and preparations

Twelve eggs from each treatment (2 eggs per replicate) were collected on the last day of the experiment. The eggs were broken and the albumen and yolk was separated and sampled. The yolks from two eggs per replicate were pooled and stored at −20°C for Mn content analysis. On E18.5, 24 embryos (4 per replicate) from each treatment were killed by cervical dislocation. Liver and heart from the embryos were immediately dissected and frozen in liquid nitrogen and then stored at −80°C for further analyses.

### Measurements of manganese contents and antioxidant indices

The Mn contents in egg yolk and the embryonic liver were determined as described previously [19]. The homogenates of embryonic liver and heart samples were prepared to determine total protein content, MDA level and SODs activities following the protocols in our previous study [57]. A commercial assay kit was used to determine either total protein concentration (Cat #23225, Pierce, Rockford, IL, USA) with the bicinchoninic acid (BCA) assay or MDA level (Cat #A003-1, Nanjing Jiancheng Bioengineering Institute, Nanjing, China) with the thiobarbituric acid assay. The TSOD, MnSOD and CuZnSOD activities were measured following the nitrite method [19]. Tissue MDA content and activities of MnSOD and CuZnSOD were expressed as one unit per mg protein.

### Gene expression analyses by RT-qPCR

Total RNA was extracted from the embryonic heart tissue using Trizol reagent (Cat #15596018, Life Technologies, Carlsbad, CA, USA) following the instructions. The cDNA synthesis was performed using QuantiTech Reverse Transcription Kit (Cat #205311, Qiagen, Chatsworth, CA, USA) following the manufacturer's protocols with genomic DNA wiping off. The protocol of two-step PCR reaction was performed by ABI 7500 Fast Real-Time PCR system following the manufacturer's instructions. Each PCR reaction was conducted for one gene of all samples in one plate. The primer sequences are listed in Supplementary Table S1. The internal references and calculations of relative gene mRNA expression for each target gene were used as described previously [58].

### Tissue preparations and western blotting

Total protein was extracted from frozen samples of the embryonic heart tissue (40 mg) using ice-cold RIPA lysis buffer (Cat #P0013B, Beyotime Institute of Biotechnology, Haimen, China) including protease inhibitor (Cat #4693159001, Roche, Penz-berg, Germany). Nuclear protein was extracted to measure the status of histone acetylation using a nucleoprotein isolation kit (Cat #P0028, Beyotime Institute of Biotechnology, Haimen, China). The protein concentrations of lysates were detected using the BCA method as mentioned above. The lysates were subjected to electrophoresis on a SDS-PAGE gel, and were then transferred onto polyvinylidene fluoride blotting membranes (Cat #IPVH00010, Merck-Millipore, Billerica, MA, USA). After transfer, membranes were incubated in blocking buffer with 5% skim milk at room temperature for 1 h and then incubated with the following primary antibodies with dilution listed in Supplementary Table S2. After 4 washes, the secondary antibody (Cat #CW0103A, ComWin Biotech, Beijing, China, diluted 1:5,000) was applied at room temperature for 1 h. The GAPDH was selected as the loading control for total protein, while H3 was selected as the loading control for nuclear proteins. The signals were recorded and analyzed with using an ECL-plus detection system.

### Global DNA methylation quantification

Total DNA was isolated from the embryonic heart tissue samples using Lysis buffer, proteinase K (Fermentas, Glen Burnie, MD, USA), and Rnase A (Qiagen, Valencia, CA, USA) followed by phenol–chloroform extraction and ethanol precipitation. The methylation of the global DNA was detected using a MethylFlash Methylated DNA Quantification Kit (Cat #P1035, Epigentek, Farmingdale, NY, USA) according to the manufacturer's instructions.

### Methylated DNA immunoprecipitation (MeDIP) analysis

The MeDIP analysis for the gene promoter was conducted as previously described [58]. Small fragments of DNA between 600 and 200 bp were prepared by sonication and denatured to produce single-stranded DNA. A portion of input DNA samples (20 ng/μL) was obtained after the denaturation. The rest was incubated with 5-methyl-cytidine antibodies (Cat #ab10805, Abcam, Cambridge, MA, 5μL). The binding complexes were immunoprecipitated with protein A/G agarose beads (50% slurry, Cat #sc-2003, Santa Cruz Biotechnology, CA, USA). The MeDIP DNA were purified with digestion buffer including proteinase K. We designed primers specific to the proximal 5’ upstream promoter sequences of *MnSOD* (-292 to-49 bp upstream from the transcription initiation site, Supplementary Figure S2A). The primer pair for *MnSOD* produced a 244 bp amplicon with 10 CpG sites (Supplementary Table S1). The real-time PCR of input and MeDIP DNA and calculation of relative changes in the extent of promoter methylation were performed as previously described [59].

### Chromatin immunoprecipitation (ChIP) analysis

The ChIP analysis for gene promoter was determined as previously described [58]. The sonicated chromatin from the embryonic heart tissue samples was prepared and precleared following the instructions of previous publication [58]. The input control, and chromatin immunoprecipitation with anti-acetylated histone 3 Lysine 9 (AcH3K9) and nonspecific IgG as a negative control (NC) were obtained after overnight at 4°C with rotation. The fragments from the input and immunoprecipitated complexes of AcH3K9 and NC were released by reverse cross-linking at 65°C for 5 h to purified DNA. The real-time PCR of ChIP and input DNA were performed using the same specific primers for *MnSOD* promoter as described in MeDIP analysis. ChIP with antibodies against AcH3K9 or IgG was used to determine the fidelity of the ChIP protocol (Supplementary Figure 2B). The values of *GAPDH* promoter region were adopted to normalized for each target gene. The fold enrichment of each target sequence was calculated using the following formula [fold enrichment=2^-(Ct AcH3K9–Ct input)^] [59].

### Statistical analyses

All data were analyzed by 2-way ANOVA using the general linear model procedure of the SAS 9.2 (SAS Institute Inc., Cary, NC), and the model included the main effects of M-TEMP, M-Mn, and their interaction, and treatment comparisons for significant differences were tested by the LSD method. Embryo mortality data were transformed to arcsine values before statistical analysis. The observation from one replicate is regarded as an experimental unit for all statistical analyses. Significant differences were set at *P* ≤ 0.05.

## SUPPLEMENTARY MATERIALS FIGURES AND TABLES



## Abbreviations

BAX: BCL2-associated X protein
BCL2: B-cell CLL/lymphoma 2
Casp3: caspase-3
CDK6: cyclin-dependent kinase 6
ChIP: chromatin immunoprecipitation
CON: control group
COX2: cyclooxygenase 2
CuZnSOD: copper zinc superoxide dismutase
GPx: glutathione peroxidase
HSP90, HSP70, HSP35 and HSP25: heat shock proteins 90, 70, 35 and 25
H3K9: histone 3 lysine 9
M-HT: maternal high temperature
M-iMn: maternal diet supplemented with inorganic Mn
MDA: malonaldehyde
MeDIP: methylated DNA immunoprecipitation
Mn: manganese
M-NT: maternal normal temperature
M-oMn: maternal diet supplemented organic Mn
ROS: reactive oxygen species
M-TEMP: maternal environmental temperature
MnSOD: manganese superoxide dismutase
iNOS: inducible nitric oxide synthase
TSOD: Total superoxide dismutase
DNMT3a and DNMT3b: DNA methyltransferases 3a and 3b
HDAC2: histone deacetylase 2
