# Supplementary material for: Maternal dietary manganese protects chick embryos against maternal heat stress via epigenetic-activated antioxidant and anti-apoptotic abilities
Source: Oncotarget. 2017 Sep 11;8(52):89665–80. doi: 10.18632/oncotarget.20804 (PMC5685700; doi:10.18632/oncotarget.20804)
Supplement: Supplementary file 1 [file oncotarget-08-89665-s001.pdf]

Maternal dietary manganese protects chick embryos against maternal heat stress via epigenetic-activated antioxidant and anti-apoptotic abilities

SUPPLEMENTARY MATERIALS

Supplementary Table 1: Nucleotide sequences of specific primers

Target gene	Gene bank accession no.	Product length (bp)	Sequence (5′-3′)	Applications
β-actin	NM_205518.1	95	F: ACCTGAGCGCAAGTACTCTGTCT<br>R: CATCGTACTCCTGCTTGCTGAT	mRNA quantification
GAPDH	NM_204305.1	128	F: CTTTGGCATTGTGGAGGGTC<br>R: ACGCTGGGATGATGTTCTGG	
HSP70	JX_827854.1	144	F: CGTCAGTGCTGTGGACAAGAGTA<br>R: CCTATCTCTGTTGGCTTCATCCT	
HSP90	NM_001109785.1	108	F: GAGTTTGACTGACCCGAGCA<br>R: TCCCTATGCCGGTATCCACA	
HSP35	HM237181.1	139	F: GGTCCCGAATGAATGCCCTTG<br>R: ACCGTTCTCCTGGCTCTTGG	
HSP25	NM_001010842.2	117	F: CCGTCTTCTGCTGAGAGGAGTG<br>R: ACCGTTGTTCCGTCCCATCAC	
MnSOD	NM_204211.1	137	F: TTCCTGACCTGCCTTACGACTAT<br>R: CCAGCGCCTCTTTGTATTTCT	
COX2	NM_001167719.1	84	F: TGTCCTTTCACTGCTTTCCAT<br>R: TTCCATTGCTGTGTTTGAGGT	
iNOS	D85422.1	66	F: CCTGTACTGAAGGTGGCTATTGG<br>R: AGGCCTGTGAGAGTGTGCAA	
GPx	NM_001163245	141	F: TTGTAAACATCAGGGGCAAA<br>R: TGGGCCAAGATCTTTCTGTAA	
Casp3	NM_204725.1	140	F: TGGCCCTCTTGAACTGAAAG<br>R: TCCACTGTCTGCTTCAATACC	
CDK6	NM001007892.2	72	F: TCAGATGTTGATCAGCTAGGAAAA<br>R: TCATTAGGCCAGTCCTCTTCTTCT	
BCL2	NM_205339.2	115	F: CAGGAGAAATCGAACAAAGGC<br>R: GATGACCGAGTACCTGAACC	
BAX	XM422067.4	69	F: CCTTGGTCTGGAAGCAGAAGA<br>R: TCCTCATCGCCATGCTCAT	
MnSOD promoter	ENSGALG00000011661	244	F: CCTGACCCTAATGGGATGG<br>R: CGGCGGGACGAGGAG	MeDIP and ChIP
GAPDH promoter	ENSGALG00000014442	76	F: CCTGGTGGATCGTGTGTCG<br>R: CACACCACAAAGGTCAGGCG	ChIP

GAPDH, glyceraldehyde-3-phosphate dehydrogenase; HSP70, HSP90, HSP35, and HSP25, heat shock proteins 70, 90, 35 and 25; MnSOD, manganese superoxide dismutase; COX2, cyclooxygenase 2; iNOS, inducible nitric oxide synthase; GPx, glutathione peroxidase; Casp3, caspase-3; CDK6, cyclin-dependent kinase 6; BCL2, B-cell CLL/lymphoma 2; BAX, BCL2-associated X protein; F, forward; R, reverse; MeDIP, methylated DNA immunoprecipitation; ChIP, chromatin immunoprecipitation.

Supplementary Table 2: Summary of the antibodies used

Antibodies	Molecular weight, KD	Host species	Source	Clonality	Catalogue no.	Dilution	Applications
GAPDH	37	Rabbit	Abcam	Polyclonal	Ab22555	1:5,000	Western blotting
HSP70	71	Rabbit	Abcam	Polyclonal	Ab69412	1:3,000	
MnSOD	26	Rabbit	Abcam	Polyclonal	Ab13533	1:3,000	
BCL2	26	Rabbit	Santa	Polyclonal	sc-492	1:100	
DNMT3a	102	Rabbit	Huaxingbio Science	Polyclonal	HX14015	1:3,000	
DNMT3b	91	Rabbit	Huaxingbio Science	Polyclonal	HX14322	1:3,000	
HDAC2	65	Rabbit	Huaxingbio Science	Polyclonal	HX9845	1:3,000	
Histone H3	15	Mouse	Huaxingbio Science	Polyclonal	HX1850	1:5,000	
5-methylcytosine		Mouse	Abcam	Monoclonal	Ab10805		MeDIP
IgG		Rabbit	Millipore		12-371		ChIP
Anti-acetyl histone H3K9	17	Rabbit	Millipore	Polyclonal	ABE18	1:3,000	Western blotting and ChIP

GAPDH, glyceraldehyde-3-phosphate dehydrogenase; HSP70, heat shock proteins 70; MnSOD, manganese superoxide dismutase; BCL2, B-cell CLL/lymphoma 2; DNMT3a and DNMT3b, DNA methyltransferases 3a and 3b; HDAC2, histone deacetylase2; KD, kilodalton; MeDIP, methylated DNA immunoprecipitation; ChIP, chromatin immunoprecipitation.

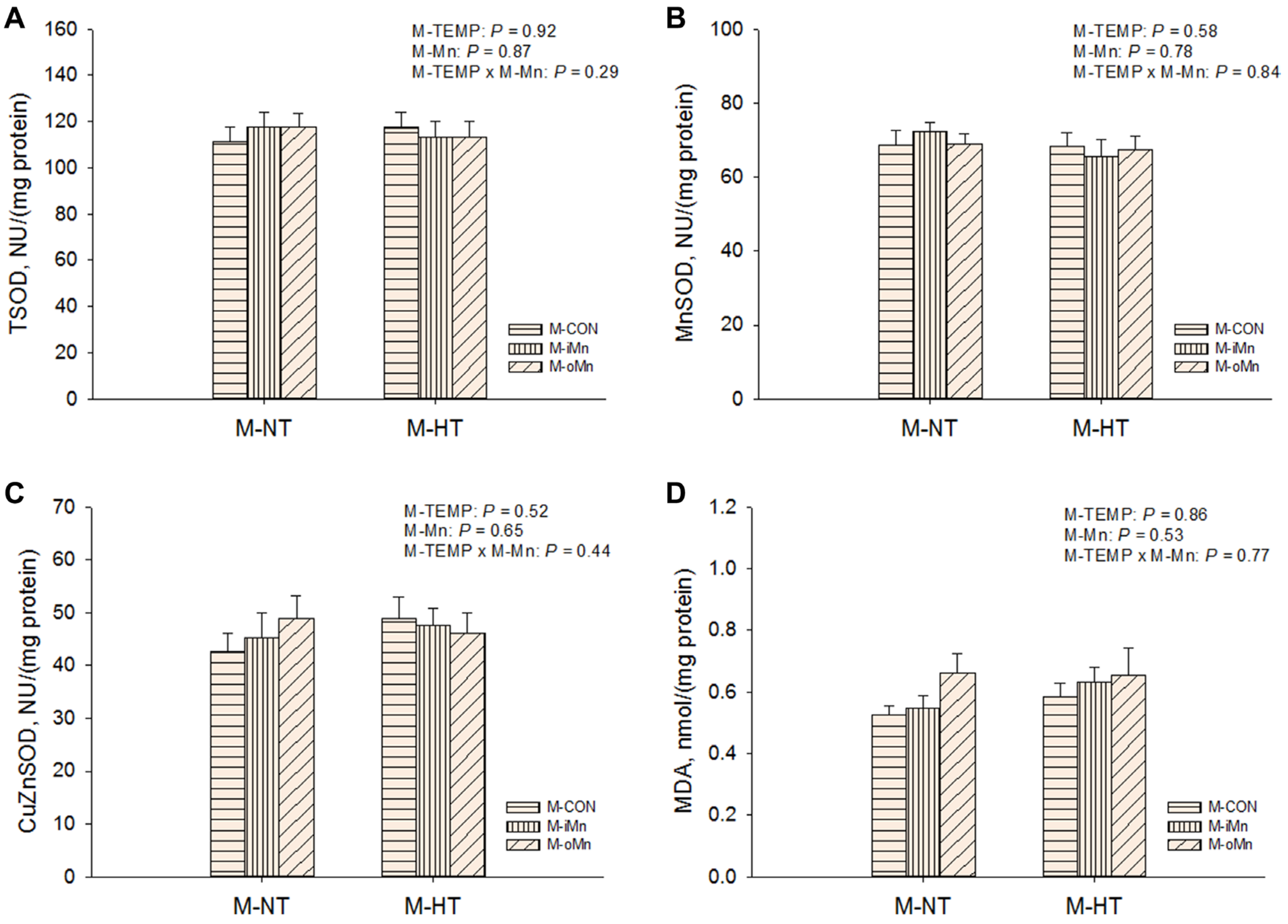

Supplementary Figure 1: Effects of maternal environmental temperature and dietary Mn on antioxidant ability in the embryonic liver. The TSOD activity (**A**), MnSOD (**B**), CuZnSOD activity (**C**) and MDA content (**D**) were used to assess antioxidant ability in the embryonic liver. The data of the above indices from the M-CON and M-iMn groups under M-NT as the control groups in the present study have been published in our previous study [31].

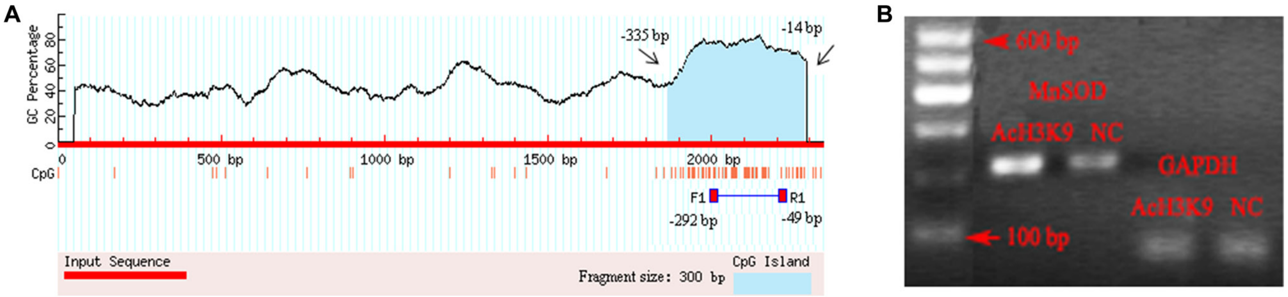

Supplementary Figure 2: The CpG-pattern rich regions in MnSOD promoter and PCR results of ChIP for H3K9 acetylation. (**A**) Schematic representations of MnSOD and the CpG-pattern rich regions were presented. Red horizontal line indicates the input sequence as a total of 2300 bp. Blue vertical area shows the position of CpG sites within the -292 bp and -49 bp fragment. (**B**) PCR results of ChIP protocol. ChIP with antibodies against AcH3K9 and rabbit IgG (NC) was used to determine the fidelity of the ChIP protocol in the embryonic heart.
